# Rectus sheath hematoma, rare condition, difficult diagnosis and multidisciplinary treatment: report of 5 cases

**DOI:** 10.1186/1471-2318-11-S1-A29

**Published:** 2011-08-24

**Authors:** Antonio Martino, Ciro De Martino, Anna Pisapia, Gautam Maharajan, Marco Evangelista

**Affiliations:** 1 Casa di Cura “A. Grimaldi” di San Giorgio a Cremano (NA). Dipartimento di Chirurgia, Italy; 2 Università degli Studi di Napoli “Federico II”. U.O.C. di Chirurgia Generale, Naples, Italy

## Background

Rectus sheath hematoma is a rare condition. It encompasses a wide spectrum of severity (self-limiting to fatal) depending on its size, etiology, and the development of complications. It has multiple possible etiologies including, frequently, coagulation disorders or anticoagulation therapy. It enters into the differential diagnosis of abdominal pain but it’s frequently difficult to diagnose and radiological imaging is often required.

## Methods

We report a series of five patients that came to our hospital within a 8-month period. The patients were between 63 to 78 years old. One of them was in therapy with warfarin, one was in therapy with acetilsaliciliate and clopidogrel and in an another patient, a coagulation disorder was detected. Diagnosis was suspected in all cases by clinical exam and ultrasonography, but CT-scan was necessary in three cases. All patients underwent conservative treatment, mainly pain relief and rest. In two cases blood transfusion was performed and in two cases clotting abnormalities were corrected with vitamin K and fresh frozen plasma. After being discharged, patients were followed up on as outpatients.

## Results

All patients were treated conservatively. Average hospitalization was 10 days (range 5-17 days). The patient healed within three months at least. One patient developed infection of the hematoma and was treated with ultrasound-guided aspiration and antibiotics.

## Conclusions

Rectus sheath hematoma is a rare but important entity in the differential diagnosis of abdominal pain. Interdisciplinary awareness of this condition is essential, as it is frequently difficult to diagnose, leading to delay in treatment or unneeded surgery. CT-scan is the gold-standard investigation. Treatment options are variable and include conservative treatment, intravascular embolization and surgery. Frequently an interdisciplinary team approach is needed.
